# The Integral Role of Physiotherapy in Optimizing Movement and Function in a Case of Polytrauma: A Case Report

**DOI:** 10.7759/cureus.61427

**Published:** 2024-05-31

**Authors:** Samruddhi Aherrao, Pratik Phansopkar, Priya Tikhile

**Affiliations:** 1 Cardiorespiratory Physiotherapy, Ravi Nair Physiotherapy College, Datta Meghe Institute of Higher Education & Research, Wardha, IND; 2 Musculoskeletal Physiotherapy, Ravi Nair Physiotherapy College, Datta Meghe Institute of Higher Education & Research, Wardha, IND

**Keywords:** ilizarov fixator, open reduction internal fixation, physical therapy rehabilitation, fracture, polytrauma

## Abstract

Multiple fractures are frequently encountered in adults following road traffic accidents. A 32-year-old male presented with multiple fractures in his right lower extremity, including a femoral shaft fracture, distal third fractures of the tibia and fibula, as well as a calcaneal fracture. The patient provided a history indicative of a road traffic accident. X-rays were performed on both hip joints, both knee joints, and the ankle joints. Treatment involved open reduction and internal fixation (ORIF) with interlocking nailing for the femur, tibia, and fibula, alongside ORIF with plating using a screw-out set (SOS) and cannulated cancellous (CC) screw fixation for the calcaneal fracture. Additionally, the Ilizarov procedure was conducted following debridement over the right foot. Post-surgery, the patient experienced primary symptoms of hip joint pain and restricted hip joint movement. Physiotherapy was initiated to address these issues. Evaluation of outcome measures indicated a reduction in joint pain, significant enhancement in joint mobility, and an increase in muscle strength.

## Introduction

Lower-extremity non-union trauma is frequent in both the military and civilian populations. Therefore, the return of a patient to their pre-disability level of function is the primary emphasis of the surgical and rehabilitation team, frequently in the face of conflicting short-term objectives [1]. In non-union surgery, impaired healing of tibial fractures continues to be a burden, impacting the quality of life and creating financial issues. To solve or prevent these issues, a complex strategy for bone healing process management and stimulation is required. According to the so-called diamond concept, vascularity, mechanical environment, osteogenic cells, osteo-inductive stimulants, and osteo-conductive scaffolds are the five elements that promote bone healing [2]. In order to minimize health risks and fatalities, patients with hip fractures typically need to be hospitalized and have surgery. The primary objectives of hip fracture surgery are rapid recovery and return to prior, notwithstanding the wide range of surgical methods available [3]. More than half of the patients still cannot return to their pre-injury level of movement, according to reports. Physiotherapists and doctors have been concerned about factors that affect functional rehabilitation [4]. In order to effectively control post-operative pain, multi-model pain treatment is now advised. Using isometric motions is one of the best ways to lessen post-operative pain in fracture patients [5]. You’ll probably lose range of motion and muscle strength in the affected area as you heal [6]. The degree and direction of the force applied determines the fracture’s geometry and deviation. These fractures can affect the medial condyle, lateral condyle, or both, and they can have a variety of fracture configurations with varying degrees of articular depression and displacement [7]. To affix subtrochanteric fractures definitely up to this point, various techniques have been used, including intramedullary nails, angled blade plates, locking plates, dynamic hip screws (DHS), dynamic condylar screws (DCS), and dynamic condylar plates. The DHS and DCS have a higher risk of reduction loss, implant failure, and the need for a general re-operation despite research showing the efficacy of each of these divisions [8].

All central fractures, except for peripheral fractures, affect the subtalar joint, which can result in different degrees of arthritis or even osteoarthritis [9]. Physiotherapy helps you feel comfortable when lying, sitting, and standing during the early phases of your recovery by reducing discomfort and swelling. A continuum of care should always manage patients with polytrauma, including the trauma site, the accident and emergency room, and any necessary damage control surgical procedures [10]. Physiotherapy is useful for treating a variety of medical conditions, including helping critically ill patients who need invasive or non-invasive non-union support, preventing respiratory, circulatory, and motor complications, and supporting critically ill patients during the pre- and post-operative periods. Early physiotherapy also involves therapeutic exercises, bedside sedation, orthostatism, armchair transfers, and ambulation for patients with a variety of functional problems [11]. The effectiveness of Ilizarov technology has been extensively established, and it is a common treatment for bone abnormalities and infected non-unions [12]. For the duration that the frame is in place, a limb stabilized in an Ilizarov external fixator needs to be used in a physiologic manner. For the correct development and ossification of either a fracture callus or a lengthening distraction gap, weight-bearing for lower extremity applications and the functional use of upper limbs are important [13]. To speed up your healing in the first week, early mobility and partial weight bearing are also crucial [14]. Regaining independence from an activity varies depending on the valued function [15]. According to some studies, only 33% and 21% of survivors, respectively, were able to perform their pre-fracture function in five fundamental and six instrumental daily life activities [16].

The purpose of this case report was to assess the outcomes and treatment implications of treating a sequential series of patients who had open reduction and internal fixation for fractures in their right leg, including the calcaneum, distal fibula, and distal third of the tibia. The subject in this case report, who was 32 years old, had fractures in the right leg's calcaneum, distal fibula, tibia, and femur shaft. Effective post-operative physical therapy was essential to minimize potential problems after open reduction and internal fixation. Previous research on treating these kinds of fractures has mostly provided a restricted understanding of physical therapy techniques and has not provided a thorough week-by-week rehabilitation schedule. To bridge this gap, an integrated physiotherapy program with weekly treatment protocols was developed and put into practice for fractures of the intertrochanteric region. This holistic approach not only enhances patient care but also serves as a valuable resource for therapists engaged in similar rehabilitative efforts, facilitating patients' restoration to their pre-injury functional state.

## Case presentation

Patient information

**Figure 1:**
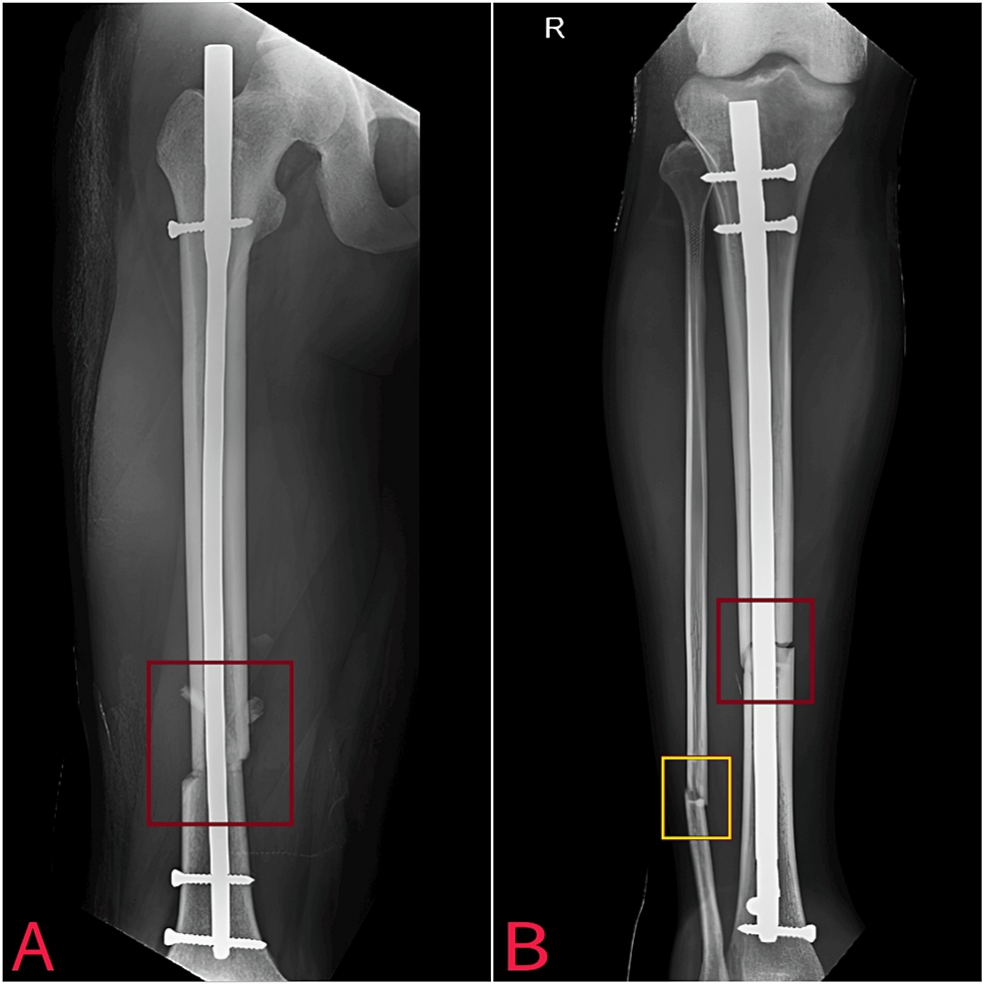
Open reduction internal fixation with the femur interlock nail for femur shaft fracture of the right side (A). Open reduction internal fixation with the tibia interlock nail for distal 1/3rd tibia fracture (B). Yellow rectangle: fractured fibula; red rectangle: fractured tibia.

In this case report, the 32-year-old participant presented with fractures in the femur shaft, distal 1/3rd of the tibia, distal fibula, and calcaneum of the right leg. Thus, the patient went through a road traffic accident and got severe fractures. Suddenly on pre-assessment, he had severe swelling edema and pain over his right side and also undergone some complications. After this, he managed conservatively for one day, and as complications reduced, he managed surgically with open reduction and internal fixation. Following open reduction and internal fixation, effective post-operative physical therapy was imperative to mitigate potential complications. On the post-assessment, he also had reduced strength, reduced range of motion, and severe pain on the numerical pain rating scale (NPRS). According to this, physical therapy management has been started. Existing literature on managing such fractures has predominantly offered limited insights into physical therapy methods, lacking a comprehensive week-by-week rehabilitation framework. In response to this gap, an integrated physiotherapy regimen featuring weekly treatment protocols was devised and implemented for intertrochanteric fractures. According to the orthopedic trauma association classification fibula is type A: A3 simple transverse fracture and tibia is type A: A1 simple spiral fracture, as shown in the X-ray in Figure 1.

As part of the patient's ongoing care plan, the patient was referred to musculoskeletal physical therapy. However, after a few days, a septic formation developed over the right foot, necessitating wound debridement. Under spinal anesthesia, thorough septic precautions were taken, including cleaning, painting, and draping of the affected area. The following day, an incision was made over the previous scar, soft tissue dissection was performed, and necrotic tissue was identified and excised. Subsequently, Illizarov fixation was conducted. Despite these interventions, physiotherapy sessions were continued as planned. The timeline of events is detailed in Table 1.

**Table 1:** The table explains the timeline of events according to the patient's condition.

Events	Date
Date of road traffic accident	21st March 2024
Dates of admission	21st March 2024
Date of surgery	22nd March 2024
Date of assessment	23rd March 2024
Date of physiotherapy rehabilitation	23rd March 2024
Date of post-assessment	4th April 2024
Date of Illizarov application	22nd April 2024

Clinical findings

Before the examination began, the patient gave their informed consent, and a comprehensive assessment was carried out. He seemed helpful and aware of their surroundings. Over the course of the assessment, his heart rate stayed constant. The patient was seen lying supine, with a cushion supporting the lower limb. The right leg showed external rotation, and there was edema around the hip. The patient responded in discomfort when palpation in the proximal tibia region exhibited grade 1 tenderness. In addition, there was a minor rise in the local temperature. The right foot showed signs of edema, although deep and superficial feelings were unaffected. The BMI of the patient was found to be 27.4 kg/m^2^. Upon examination, the right leg's quadriceps muscle wasting was discovered. Applying the numerical pain rating scale (NPRS), the patient reported pain levels of 9/10 during movement and 5/10 at rest.

Physiotherapy intervention

Table 2 presents a detailed, goal-oriented advanced physical therapy rehabilitation program for post-operative open reduction internal fixation with femur interlock nailing for femur shaft, tibia, and fibula fracture with a compound grade 3B calcaneum fracture of the right foot.

**Table 2:** Advanced physical therapy intervention according to the patient's condition. Thus, the table explains the intervention with the dosage, the timeline, and the rationale. The week-wise treatment has been discussed. NA: not assessable; ROM: range of motion.

Time	Goal	Intervention	Rationale	Dosage
Week one	Patient education	Educating the patient about his ailment and the planned physiotherapy procedure.	Education equips the patient with the ability to play a proactive part in their recovery journey and guarantees adherence to the rehabilitation regimen.	NA
	Control pain begins to restore ROM	Cryotherapy involves applying an ice pack to a sore location for 8-10 minutes. Ankle pumps	To help manage pain, reduce swelling, and promote healing.	10 Reps with 10 s hold one set
	Restore independent functional mobility	Functional mobility-bed mobility transfer training. From supine to side lying, from side lying to sitting, from sitting to supported non-weight bearing standing. Then gait training should be initiated.	To help patient, regain proper walking patterns, restore balance and stability, and facilitate a safe return to normal mobility and activities of daily living.	NA
Week two	Begin to restore muscle strength	Isometrics for quadriceps by putting a rolled-up towel beneath the knee and hold the contraction for 10 s; Isometrics for Hamstring putting a rolled-up towel beneath the heel and hold the contraction for 10 s; Isometrics for gluteus including the gluteus maximus, medius, and minimus.	For preventing muscle atrophy, promoting blood circulation, and supporting the healing process.	10 Reps with 10 s hold one set; 10 Reps with 10 s hold one set; 10 Reps with 10 s hold one set
	To improve upper limb strengthening	Strengthening exercises are given with theraband and dumbbells for both upper limbs	Strengthening the upper limbs enhances stability, posture, and balance, crucial for proper walking mechanics. It also aids coordination and supports weight transfer, improving gait efficiency.	10 Reps with 10 s hold one set
Two to three weeks	To restore muscle strength throughout the operated leg	Strengthening with theraband; Dynamic quadriceps exercises with holds	Building strength through theraband is crucial for recovering muscle function and reducing the risk of future injuries.	10 Reps with 10 s hold one set; 10 Reps with 10 s hold one set
	To improve ROM	Hip flexion, abduction, and adduction while standing. Progress to SLRs, hip abduction, adduction, and extension against gravity.	Starting passive range of motion (ROM) exercises early can prevent stiffness and keep joints mobile after surgery. As pain allows, the therapist can introduce active ROM exercises to encourage the swift restoration of active movement.	10 Reps with 10 s hold one set
	To improve strength	Closed chain exercises terminal knee extension, mini-squats mini-lunges	This will engage multiple muscle groups, improve joint stability, and enhance functional movement patterns, promoting a safe and effective recovery.	10 Reps with 10 s hold one set
Four to six weeks	To improve proprioception	Proprioception weight shifting activities; single leg stance	It helps restore joint stability, enhances coordination, and improves balance, ultimately reducing the risk of re-injury, and facilitating a smoother return to daily activities.	Two times a day
Six weeks	To improve endurance	Progressive resistance exercises are given. Progress mechanical resistance with therabands and weight cuffs	It aids in overall recovery and enabling individuals to regain their ability to engage in daily activities and return to their desired level of physical function.	Two times a day
Seven weeks	Return to baseline functional activities	Resisted training with theraband or weight cuff. Resisted hip flexion, extension, abduction, and knee extension. Proprioception starts with a single-leg stance, static balance on foam, light agility exercises (e.g., tandem walking, side stepping, and backward walking), and a walking program for endurance.	This helps rebuild strength in muscles and tissues that may have weakened due to injury and is essential for supporting joint stability and facilitating a successful recovery process.	10 Reps with 10 s hold one set; 10 reps with 10 s hold one set; 10 reps with 10 s hold one set; 10 reps with 10 s hold one set
Six to eight weeks	To improve gait training	Partial weight-bearing gait training should be initiated in which the operated leg should partially touch the ground and the non-operated leg should fully be on the ground surface.	Correct walking techniques, re-establishing equilibrium and steadiness, and enabling a secure resumption of regular mobility and everyday tasks.	Two rounds over a 50 m hallway
Nine weeks	To improve the functional mobility	When the patient is allowed for weight bearing then the patient can perform ambulatory transfers from bed to chair with assistance. A raised toilet seat is used to decrease hip flexion and reduce stress at the fracture site.	It ensures that patients can safely and comfortably manage their toileting needs, contributing to their overall recovery, regain independence and quality of life.	NA
Beyond eight weeks	Agility training	Multidirectional stepping weight shifting side-stepping side shuffle grape wine figure of 8 walking. Backward walking cone agility and technique drill.	For enhancing dynamic movement, coordination, regain functional mobility, adaptability, and confidence in their physical abilities, which are essential for safely navigating daily activities and returning to sports or recreational pursuits.	NA

Follow-Up and Outcome Measures

Table 3 shows outcome measures.

**Table 3:** Outcomes measures like visual analog scale, numerical pain rating scale, time up and go test, lower extremity functional scale, and quality of life were taken.

S. no	Outcomes	On first day	On week two
1	Visual analog scale	7/10	3/10
2	Numerical pain rating scale	9/10	3/10
3	Time up and go test	<30 s = cannot go outside alone, requires gait aid	<10 s = normal mobility achieved with support
4	Lower extremity functional scale	9/100	48/100
5	Quality of life	35-50 complete disability	5-14 mild disability

After eight weeks of rehabilitation, the patient may be able to perform all activities of daily living (ADLs) and may not have any pain or discomfort. She was happy with the recommended course of action and could perform daily duties with ease, which eventually raised her quality of life (QOL). The range of motion pre- and post-treatment are displayed in Tables 4, 5. Manual muscle testing is shown in Table 6.

**Table 4:** Pre-treatment range of motion according to patient's condition. NA: not assessable

Joint/movement	Quality of movement	Right (active)	Right (passive)	Normal ROM	Left (active)	Left (passive)	End feel
Hip flexion	Grade 4	0-60	0-70	0-120	0-85	0-90	Empty
Hip extension	NA	NA	NA	0-30	NA	NA	NA
Hip abduction	Grade 3	0-10	0-15	0-25	0-20	0-25	Empty
Hip adduction	Grade 3	0-10	0-15	0-30	0-20	0-25	Empty
Knee extension	Grade 3	NA	NA	0-135	NA	NA	NA
Ankle dorsiflexion	Grade 3	NA	NA	0-20	0-15	0-15	Firm
Ankle plantarflexion	Grade 3	NA	NA	0-35	0-30	0-30	Firm

**Table 5:** Post-treatment range of motion according to patient's condition.

Joint/movement	Quality of movement	Right (active)	Right (passive)	Normal ROM	Left (active)	Left (passive)	End feel
Hip flexion	Grade 4	0-80	0-80	0-120	0-85	0-90	Empty
Hip extension	NA	NA	NA	0-30	NA	NA	
Hip abduction	Grade 4	0-15	0-20	0-25	0-20	0-25	Empty
Hip adduction	Grade 2	0-10	0-15	0-30	0-20	0-25	Empty
Knee flexion	Grade 2	0-70	0-90	0-135	0-100	0-100	Empty
Knee extension	Grade 3	NA	NA	0-135	NA	NA	
Ankle dorsiflexion	Grade 3	NA	NA	0-20	0-15	0-15	Firm
Ankle plantarflexion	Grade 3	NA	NA	0-35	0-30	0-30	Firm

**Table 6:** Manual muscle testing according to patient's condition.

Muscles	Left	Grade	Right		Pre-grade	Post		Grade
Hip flexors	4/5	Holds test position against slight pressure	2+/5	Moves through a partial range of motion		3/5	Holds test position (no added pressure)	
Hip extensors	2/5	Moves through the complete range of motion	1/5	Feeble contraction feels in the muscle, but no visible movement of part		3/5	Holds test position (no added pressure)	
Hip abductors	4/5	Holds test position against slight pressure	2+/5	Moves through a partial range of motion		3/5	Holds test position (no added pressure)	
Knee flexors	4/5	Holds test position against slight pressure	2/5	Moves through a complete range of motion		3/5	Holds test position (no added pressure)	
Knee extensors	3/5	Holds test position (no added pressure)	3/5	Holds test position (no added pressure)		4/5	Holds test position against slight pressure	
Ankle dorsiflexors	5/5	Holds test position against strong pressure	2+/5	Moves through a partial range of motion		4/5	Holds test position against slight pressure	
Ankle plantarflexors	5/5	Holds test position against strong pressure	1/5	Feeble contraction feels in the muscle, but no visible movement of part		4/5	Holds test position against slight pressure	

## Discussion

Following hip fractures, femur fractures are the most common reason for morbidity, hospitalizations, and death among the elderly. Additionally, a significant portion of these individuals never restore their pre-fracture functional status [8]. Clinically and radiologically, hip fractures are frequently easy to detect, with initial imaging sensitivity estimated to be 90-98%. Every year in the UK, about 75,000 femoral neck fractures occur, and this number is expected to continue to climb [17]. After hip trauma, occult fractures-which are invisible on standard radiography-are thought to happen in 2% to 10% of all pelvic and hip joint locations. Femoral neck, ITC, and pelvic pubic rami fractures are a few examples of these fractures [18].

It has been demonstrated that the healing of tibia and fibula fractures is better when progressive range-of-motion exercises, soft tissue recruitment, isometric exercises, open- and closed-chain muscle-strengthening activities, stretching, and training for gait are used [19]. Early physical therapy intervention following a hindfoot fracture has been shown to improve long-term results and reduce complications [20]. The muscle energy technique is a type of manual treatment that helps with muscle strength and range of motion. In tough post-operative situations, physiotherapy can assist in maintaining and enhancing strength and mobility. Maintaining muscle integrity while increasing lower assistance [21]. Previously, arthroscopic-assisted surgical methods were frequently employed because they make intra-articular lesion diagnosis easier. Furthermore, arthroscopic-assisted procedures have a low risk of morbidity and are minimally invasive. Indeed, MRI and arthroscopic aided procedures are helpful in the diagnosis of soft tissue injuries [22]. According to Gabriel's studies, gait and confidence are enhanced with post-operative physiotherapy. Given is a muscular energy technique that assists with lower extremity range of motion, pain reduction, and increased strength and flexibility. Physiotherapy can support the maintenance and enhancement of strength and mobility in situations of traumatic post-operative recovery [23]. Physiotherapy can help you recover from your injury more quickly and with a better prognosis [24]. Mobility, ambulation, and self-care are all greatly impacted by a hip fracture. It has been demonstrated that this decline in quality of life lasts for several years [25]. Based on the available data, it appears that the LEFS is a valuable tool for documenting lower-extremity activity, particularly given its ease of administration and scoring. However, only prospective studies will establish the utility of this biomarker in clinical decision-making. We believe that a generic health status measure such as the SF-36 should be included in the LEFS to allow us to assess our patients' general wellness state, given the LEFS only evaluates physical function [26]. In clinical and research contexts, the TUG is often used to identify individuals who are at a higher risk of falling. The frequently mentioned cutoff point of ≥13.5 seconds is used to determine who is most in danger of falling and assists patients in determining the length of time they can execute the [27]. A skilled musculoskeletal physiotherapist provided the patient with a comprehensive physical therapy rehabilitation program that included several activities and resistive equipment, according to the most recent report. Cryotherapy and opioids provided significant pain relief, allowing the patient to focus more on their rehabilitation, which improved their joint movement, strength training, and clinical status [28]. The purpose of the physical therapy sessions was to keep the muscles in the right lower leg intact while also enhancing the function of the left lower limb and both upper limbs. This would allow each patient to walk independently with the help of an assistive device and require minimal assistance with daily duties. In order to successfully supervise the patient, this case study emphasizes the importance of timely reconstructive surgery, the necessity of physical rehabilitation and physical therapy services, and the offering of an integrated protocol with weekly treatment plans. Thus, the result obtained is a functional recovery, pain management, bone union, and a reduction in complications by improving balance training with stabilization and also improving physical endurance and strength with joint mobility. 

## Conclusions

The post-fracture treatment program has proven productive, leading to significant enhancements in both well-being and physical functionality. The case report outlines a comprehensive treatment plan for patients who have undergone fracture repair recovery. Following eight weeks of focused physical therapy, the patient experienced improved muscle strength, a gradual increase in hip range of motion, enhanced functional capacity, reduced pain, enhanced ambulation, and better performance in routine tasks. While full recovery wasn't achieved within the treatment period, most of the restorative goals were met.
